# Chronic lead poisoning induced abdominal pain and anemia: a case report and review of the literature

**DOI:** 10.1186/s12876-020-01482-x

**Published:** 2020-10-14

**Authors:** Yuan Yang, Shujun Li, Hong Wang, Morong Liu, Biguang Tuo, Huichao Wu, Shili Deng, Xuemei Liu

**Affiliations:** 1 Department of Gastroenterology, Affiliated Hospital of Zunyi Medical University, Zunyi, 563003 Guizhou, Province China; 2 Department of Burn and Plastic Surgery, Affiliated Hospital of Zunyi Medical University, Zunyi, 563003 Guizhou, Province China

**Keywords:** Chronic lead poisoning, Abdominal pain, Anemia, Differential diagnosis

## Abstract

**Background:**

Chronic lead poisoning (CLP) is a rare cause of abdominal pain and is common in young children, in whom the incidence is higher than it is in adults. As the symptoms of CLP are nonspecific, misdiagnoses or missed diagnoses often occur, especially in sporadic cases.

**Case presentation:**

We report a 28-year-old young man who was misdiagnosed with renal colic due to sudden acute abdominal pain. After a detailed medical history and physical examination, other possible causes were excluded, CLP was finally diagnosed, and he recovered after chelation treatment.

**Conclusion:**

Abdominal pain is a very common clinical symptom in adults, which has many causes. We should be vigilant against chronic poisoning, especially CLP. Detailed diagnosis and physical examination are crucial in early diagnosis and treatment.

## Background

Abdominal pain is a very common clinical symptom in adults. Many etiologies can cause abdominal pain that lacks specificity, which is easy to ignored in diagnosis or misdiagnose. Chronic lead poisoning (CLP) is a rather rare etiology of abdominal pain, and is common in young children, in whom the incidence is higher than it is in adults. Lead is the most important toxic heavy element in the environment, due to its important physico-chemical properties, it is applied widely, which has led to environmental pollution in different areas [1]. Occupational lead poisoning is still the most common chronic poisoning in China, Characteristics of obvious occupational exposure and group morbidity can help in the diagnosis of occupational lead poisoning. However, living lead poisoning is only occasionally discovered. Here, we report an adult case of CLP caused by long-term exposure to children’s picture books, manifesting as abdominal pain and anemia, and he recovered after chelation treatment.

## Case presentation

A 26-year-old male patient was admitted to our hospital due to recurrent abdominal pain for 3 months and aggravated for 2 days. This kind of paroxysmal colic was around the umbilical and hypogastric region and radiated to the lower back. The local community hospital he once visited considered “kidney stones”. The patient was given regular symptomatic solutions, which included antispasmodic and analgesic treatments. His abdominal pain was not significantly relieved after treatment. For further diagnosis and treatment, he was sent to our hospital. Upon physical examination, his vital signs were stable. He had an anemic appearance and slight tenderness around the umbilicus during abdominal palpation, without renal percussive pain. The clinical laboratory results of the patient showed that he was moderately anemic, with normocytic anemia and elevated bilirubin, mainly indirect bilirubin, there were no obvious abnormal in other laboratory results (Table 1). Total abdominal computed tomography (CT) showed kidney stones in both sides and a small amount of pelvic effusion, and abdominal cavity and small retroperitoneal lymph nodes were increased in size. Gastroscopy showed chronic non-atrophic gastritis with bile reflux and colonoscopy was normal.

**Table 1:**
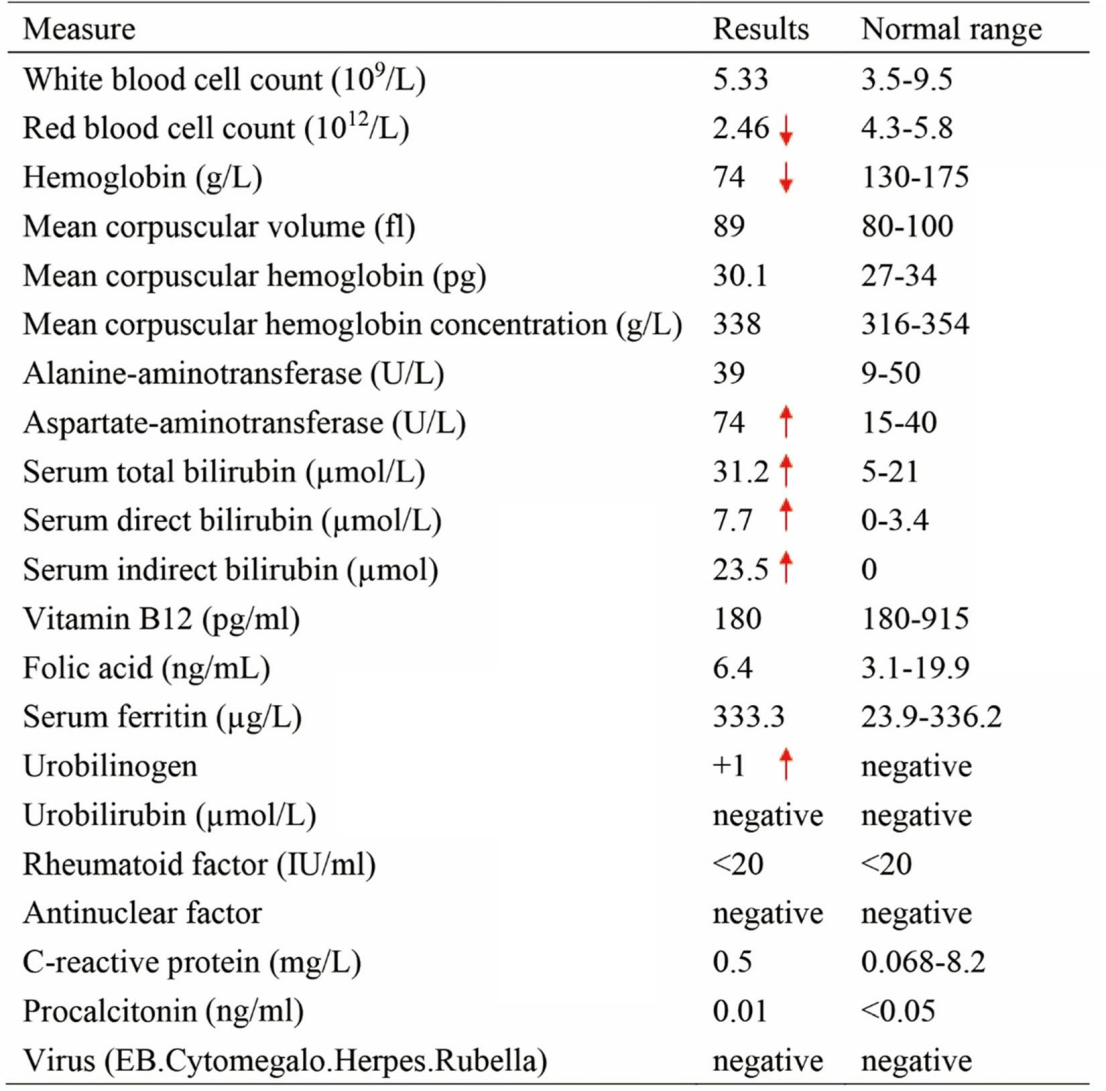
The clinical laboratory results of the patient

**Fig. 1:**
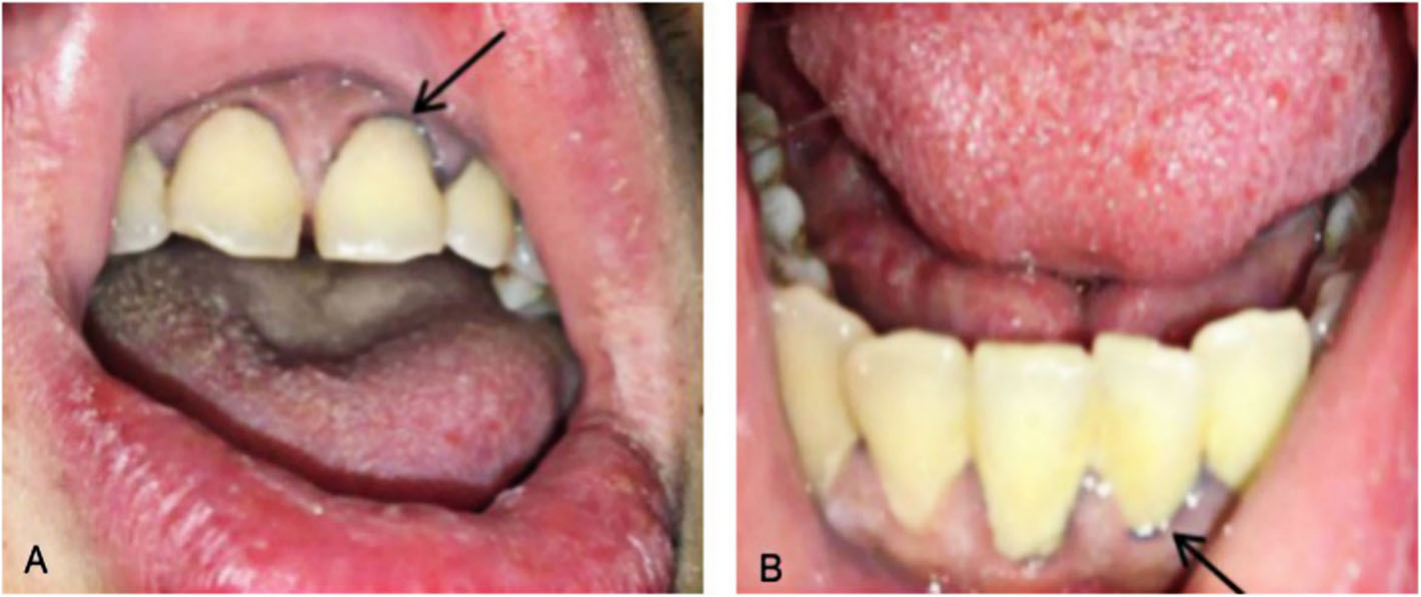
Physical examination character of CLP. **a** Burton line in the upper gum (black arrow). **b** Burton line in the lower gum (black arrow)

**Fig. 2:**
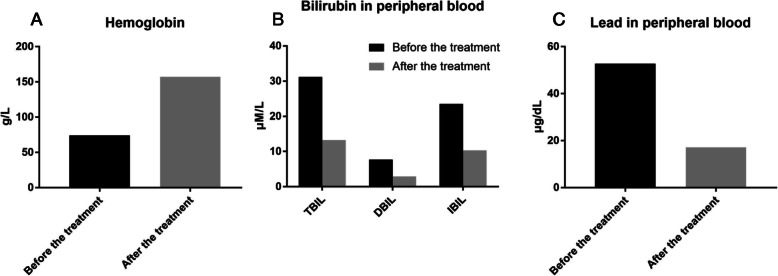
Blood examination of this case. **a** Hemoglobin concentrations in the peripheral blood 1 month before and after the treatment. **b** Bilirubin concentrations in the peripheral blood 1 month before and after the treatment. **c** Blood lead concentrations in the peripheral blood 5 months before and after the treatment

**Fig. 3:**
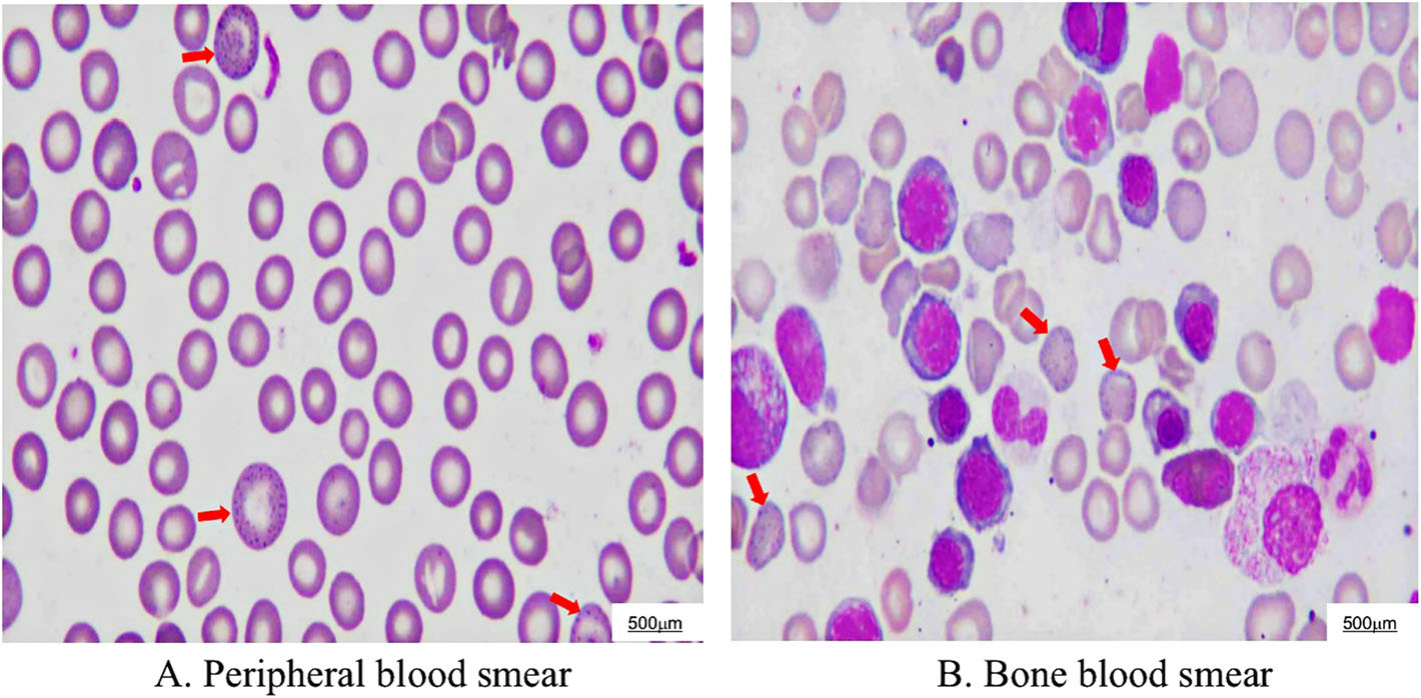
Hematological manifestations of CLP. **a** Basophilic stippling of erythrocytes in a peripheral blood smear (red arrows). **b** Basophilic stippling of erythrocytes in a bone marrow smear (red arrows)

During his hospitalization, the abdominal pain was aggravated, but some acute abdominal diseases, including pancreatitis, cholecystitis and appendicitis were excluded, based on the normality of inflammatory indicators and CT examination. Kidney stones and abdominal allergic purpura could not reasonably explain this patient’s pain symptoms, suggesting that there must be other etiology. Upon inquiring into the patient’s medical history in detail, it was learned that the patient was a warehouse keeper who had been working in the children’s new book warehouse for more than 2 years, meaning he has been in close contact with new children’s picture books for a long time. Physical examination revealed Burton’s lines over the gums, which is a classical feature of CLP (Fig. 1). Moreover, blood examination showed that his blood lead level had increased to 52.8 μg/dl (normal range < 10 μg/dl) (Fig. 2). Hematological manifestations of heavy metal poisoning and basophilic stippling of erythrocytes are found in marrow and blood smear (Fig. 3). Therefore, CLP was highly suspected. He immediately received dimercaptosuccinic acid (2,3-dimercaptosuccinic acid, DMSA) therapy 10 mg kg^− 1^ by mouth every 8 hour, taking the drug on first third days of the week, with 1 week as a course of treatment. During the first week of hospitalization, supportive treatment with water-soluble vitamins was also given. His abdominal pain was relief significantly within 2 days after treatment. After 1 month treatment, Hemoglobin (Hb) was increased from 74 g/L to 157 g/L and serum total bilirubin (TBil) was decreased from 31.2 μm/L to 13.2 μm/L, Blood lead was reduced from 52.8 μg/dl to 17.2 μg/dl in 5 months (Fig. 2). Above all indictors were normal after 8 months treatment.

## Discussion and conclusion

Abdominal pain is one of the main reasons for seeking medical care in emergency and gastrointestinal departments. Abdominal pain ranges from mild self-limiting conditions to life-threatening emergencies. It is believed that 20–40% of abdominal pain etiologies remain unknown at the time of discharge. Some patients receive unnecessary treatment, even including emergency exploratory laparotomy. The reasons for abdominal pain are mainly caused by abdominal organ diseases, but extra-abdominal diseases and systemic diseases can also cause pain; in particular, abdominal pain caused by systemic diseases is easy to ignore, such as chronic poisoning, diabetic ketoacidosis, and allergic purpura. The final correct diagnosis depends on a comprehensive analysis of a detailed medical history, comprehensive physical examination and auxiliary examination.

CLP has a long history of becoming a public health problem and is more commonly found in children than adults. The absorption of lead mainly occurs in the respiratory and digestive tracts [2]. Although the lead pollution has decreased, lead exposure shows obviously regional differences in China. Some reports of lead poisoning in some economically backward rural areas are mainly from traditional Chinese folk treatment [3]. In 2012, the United States Centers for Disease Control and Prevention increased the standard of blood lead for adults to 10 μg/dL and for children to 5 μg/dL [4]. The symptoms of CLP are nonspecific, symptoms of CLP are related to blood lead levels. Patients with mild CLP (blood lead 10 μg/dL) present common nonspecific symptoms that usually include discomfort, anorexia, abdominal pain and irritability. Extremely high blood lead levels (> 70 μg/dL) may cause cerebral edema, encephalopathy and confusion, drowsiness, coma or epilepsy, and even death [5]. The most important initial management for CLP patients is removal from the source of exposure. If blood lead levels exceed 45 μg/dl, chelation treatment is recommended. The available agents include DMSA, dimercaprol, ethylene diamine tetra-acetic acid (CaNa_2_EDTA), and D-penicillamine [6].

This patient’s abdominal pain was severe colic, but with only slight physical signs. The patient presented the kind of characteristics of abdominal pain caused by CLP. Lead is an electropositive metal with high affinity for sulfhydryl groups and thus inhibits sulfhydryl-dependent enzymes. In particular, lead also changes the vasomotor action of smooth muscle due to its effect on Ca-ATPase, which can cause abdominal pain [7]. The patient was moderately anemic, with normocytic anemia and elevated bilirubin, mainly indirect bilirubin, without evidence of hemorrhagic anemia and hematopoietic dysfunction, without supplementation of hematopoietic materials after chelation therapy, the patient’s Hb increased to normal levels. First, lead inhibits the major enzymes involved with heme synthesis of δ-aminolaevulinic acid dehydratase, coproporphyrinogen oxidase and ferrochelatase as well as pyrimidine 5′-nucleotidase, Second, lead can also be attached to the erythrocyte membrane to interfere with Na^+^-K^+^-ATP enzyme, and as a result, erythrocytes become easier to hemolyze. Finally, anemia occurs, and bilirubin levels increase [8]. The patient’s physical examination revealed the formation of Burton’s lines on the upper and lower gums; Burton’s lines are blue-purplish lines on the gums. They are caused by a reaction between circulating lead with sulfur ions released during oral bacterial activity, which deposits lead sulfide at the junction of the teeth and gums [9]. The normal features of CLP include abdominal pain, anemia with basophilic stippling of red cells, blue-black gum deposits, and a lead line on joint radiography [10]. This case reminds us that in the face of a patient with abdominal pain, we should not only consider the usual reasons for abdominal pains, but also carefully inquire about the patient’s medical history and consider some other rare etiologies, such as CLP.

In conclusion, CLP is not a common cause of adult abdominal pain, the diagnosis of CLP is often delayed, and the abdominal pain can more easily conceal the underlying illnesses, detailed history taking and physical examination are crucial in early diagnosis and treatment. This report indicates that CLP should be considered as a differential diagnosis in cases of unexplained abdominal pain when other common causes have been excluded. The diagnosis of CLP is easy once it is suspected, this study may provide more clinical experience for diagnosis of CLP.

## Data Availability

The data of this study are available from the corresponding author upon reasonable request.

## Abbreviations

CLP: Chronic lead poisoning
Hb: Hemoglobin
CT: Computed tomography
TBil: Serum total bilirubin
DBil: Serum direct bilirubin
IBil: Serum indirect bilirubin
DMSA: Dimercaptosuccinic acid
CaNa_2_EDTA: Ethylene diamine tetra-acetic acid
